# The amplitude of small eye movements can be accurately estimated with video-based eye trackers

**DOI:** 10.3758/s13428-021-01780-6

**Published:** 2022-04-13

**Authors:** Marcus Nyström, Diederick C. Niehorster, Richard Andersson, Roy S. Hessels, Ignace T. C. Hooge

**Affiliations:** 1 Lund University Humanities Lab, Lund University, Box 201, SE–221 00 Lund, Sweden; 2 Department of Psychology, Lund University, Box 201, SE–221 00 Lund, Sweden; 3 Tobii Pro AB, Box 743, SE–182 17 Danderyd, Sweden; 4 Experimental Psychology, Helmholtz Institute, Utrecht University, Heidelberglaan 1, 3584 CS Utrecht, The Netherlands

**Keywords:** Microsaccades, EyeLink 1000 plus, Image resolution

## Abstract

Estimating the gaze direction with a digital video-based pupil and corneal reflection (P-CR) eye tracker is challenging partly since a video camera is limited in terms of spatial and temporal resolution, and because the captured eye images contain noise. Through computer simulation, we evaluated the localization accuracy of pupil-, and CR centers in the eye image for small eye rotations (≪ 1 deg). Results highlight how inaccuracies in center localization are related to 1) how many pixels the pupil and CR span in the eye camera image, 2) the method to compute the center of the pupil and CRs, and 3) the level of image noise. Our results provide a possible explanation to why the amplitude of small saccades may not be accurately estimated by many currently used video-based eye trackers. We conclude that eye movements with arbitrarily small amplitudes can be accurately estimated using the P-CR eye-tracking principle given that the level of image noise is low and the pupil and CR span enough pixels in the eye camera, or if localization of the CR is based on the intensity values in the eye image instead of a binary representation.

## Introduction

Video-based eye trackers were introduced more than 50 years ago (Merchant, 1969; Merchant et al., 1974), and are today used by eye movement researchers across a wide range of fields. Typically, gaze direction is estimated by mapping the centers of the pupil and the corneal reflection as they appear in the eye image to gaze positions in the world. We refer to this method as pupil and corneal reflection (P-CR) eye-tracking (cf. Merchant et al., 1974, for an introduction to this method). A large reason for the success of P-CR eye trackers over analogue techniques such as scleral search coils (Collewijn, 1999) and the dual Purkinje eye tracker (DPI) (Crane & Steele, 1985) is that they are easier to use and maintain (compared to the DPI), and are less invasive (compared to coils). However, both coils and the DPI are generally considered to provide data of higher quality (cf. McConkie, 1981; Holmqvist et al., 2012; Hessels et al., 2017, for a more comprehensive description of data quality), and to be more suitable for estimating small amplitude eye movements such as microsaccades (Collewijn & Kowler, 2008; Poletti & Rucci, 2016).

Recently, using an artificial eye that was rotated in small steps (≪ 1 deg), (Holmqvist & Blignaut, 2020) reported that small eye movements are not accurately represented in gaze-position signals recorded from a number of commercial P-CR eye trackers. They claimed that the errors were mostly due to mis-localization of the CR center in the eye image. In this paper, we will address this claim using a simulation approach, and investigate if and under what circumstances such mis-localization occurs.

There are several practical reasons why the data from a P-CR eye tracker is limited in quality. An inherent limitation of P-CR eye trackers is that the image sensor in a video camera has a limited spatial (number of pixels) and temporal (frames per second) resolution, which poses limits in how well small and fast eye movements can be captured. In terms of spatial resolution, Mulligan (1997) argued that an eye rotation leading to the translation of one of the edge pixels in a pupil with a diameter of 200 pixels, would correspond to a displacement of the pupil center of 0.003 pixels in the eye image. This would typically correspond to a very small (≪ 1 deg) eye rotation in a real eye-tracker setup. Recently, Ivanchenko et al., (2021) described a P-CR eye-tracker setup where a pupil with diameter 4 mm would correspond to an area of 20000 pixels (diameter about 160 pixels) and where the area of the CR was 400 pixels (diameter about 23 pixels) in the eye camera. They argue that that this setup would be able to resolve eye movements as small as 3.5 min arc. However, these theoretical predictions are complicated by the fact that real eye images always contain noise, i.e., undesired variation in pixel intensity, which may significantly reduce the localization accuracy of the pupil and CR centers in the eye image. This *image noise* can originate from several different sources such as photon shot noise and quantization noise (Irie et al., 2008).

Another factor that influences how accurately an image *feature* such as the pupil or the CR can be localized is the specific method used to calculate its center. In the example by Mulligan (1997) above, the pupil center was computed as the center-of-mass of the pupil area, identified after applying a specific threshold to the eye image. Such a thresholding operation turns the gray scale eye image captured by the camera into a binary image where the pupil area is represented by black color and other regions by white color. Since the pupil typically is darker and the CR typically brighter than other parts of the eye image, thresholding is an efficient method to separate and isolate these image features from the background. Shortis et al., (1994) describe and evaluate a number of methods used to localize isolated image features, and distinguish between methods where the input is binary and where it is intensity-based. Binary methods include computing the center-of-mass or fitting an ellipse to a binary feature whereas intensity methods consider the variation in pixel intensities within the feature when computing the center-of-mass. In general, Shortis et al., (1994) conclude that intensity-based methods provide higher localization accuracy than binary methods, but that the advantage becomes smaller as the size of the feature increases.

One method to assess how accurately a small eye movement can be estimated by an eye tracker is to rotate an artificial eye in very small steps and observe if these steps can be detected in the gaze signal (Crane and Steele, 1985). While this was common practice for analogue eye trackers in the past, it is less commonly used for video-based eye trackers (Poletti & Rucci, 2016). Using an artificial eye, Holmqvist and Blignaut (2020) reported that many commonly used video-based eye trackers could record small eye movements. However, changes in eye orientation with the same angle yielded changes with different magnitudes in the gaze position signal. Based on this, Holmqvist and Blignaut (2020) concluded that while P-CR eye trackers typically can be used to detect small saccades, they are not suitable to estimate properties of the saccades such as amplitude. Specifically, they suggested that inaccurate sub-pixel estimation of the CR center was the source of this problem, but also argued that the resolution of the eye camera does not “matter in and by itself”. Even though other hypotheses as to why these problems occur were provided by Holmqvist and Blignaut (2020), they emphasize that a “A major unsolved issue is exactly what it is that causes the errors in the CR signal in modern VOGs”.^1^

If one wants to further investigate the causes behind the issue (Holmqvist & Blignaut, 2020) report, it is necessary to go beyond using commercially sold video-based eye trackers, which can be thought of as ‘black boxes’, where the details of the hardware and software implementations are proprietary (Poletti & Rucci, 2016). Since the origin of the problems (Holmqvist & Blignaut, 2020) describe reside inside the black box, it may be difficult to uncover the exact causes of these problems without having access to specific information about the eye tracker; for instance about the eye images, the algorithms used to determine the center of the pupil and CRs, and the specific model used to estimate gaze. Therefore, we will in this paper model how eye movements are recorded with a camera and how eye images are processed with a fully transparent eye-tracker setup where image noise and other challenging problems related to the localization of the pupil and the CR can be modeled under fully controlled conditions.

This paper addresses the following questions: 1) What are the limitations of estimating small eye movements with a video-based P-CR eye tracker? and 2) is localization of the CR the main issue? The second research question directly addresses the claim by Holmqvist and Blignaut (2020). We consider the problem of precisely and accurately localizing the centers of the pupil and, in particular, the CR in an eye image, which is a prerequisite for recording precise and accurate eye-tracker signals using a P-CR eye tracker. We approach this problem by simulating how the pupil and CR are projected on the image sensor, and investigating how the size of the projected pupil/CR, the amount of noise in the image, and the method used to localize the pupil/CR center influence the localization accuracy. In essence, what we do is to simulate small eye rotations (corresponding to subpixel steps of the pupil/CR on the image sensor) and use a model eye-tracker setup where relevant parameters can be systematically varied, and the output observed (Section “Simulations”). For some sets of parameters, we find substantial errors in localization accuracy of the pupil and CR. To investigate what magnitude of error to expect in a commonly used commercial eye tracker (EyeLink 1000 Plus) and in our self-built eye tracker known as the FLEX (Hooge et al., 2021), the simulations from Section “Simulations” are repeated using parameters estimated from two participants recorded with these setups.

## Simulations

**Fig. 1:**
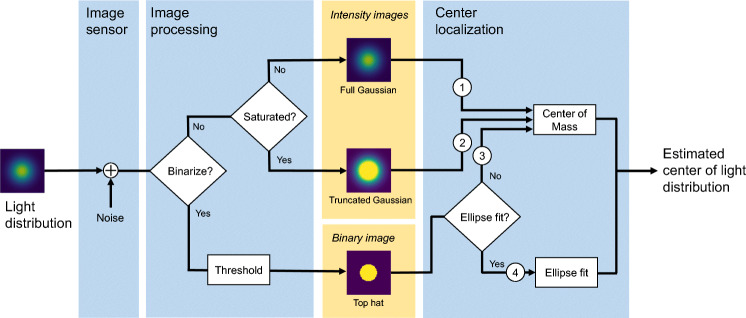
Flowchart of how the center of a light distribution from a pupil or a CR is estimated. A light distribution is digitized at the image sensor, where noise is added to the digitized image. The image is subjected to thresholding to produce a binary ‘Top hat’ image (*bottom path*), which represents a pupil or a CR in an eye image after thresholding. The intensity images represent the light distribution where the maximal brightness value can be represented on the image sensor (full Gaussian) or where the image sensor is saturated (truncated Gaussian). The centers of the pupil and CR in these images are localized either by the center-of-mass (CoM) (paths ①-③) or by fitting an ellipse to the contour points of the Top hat (path ④). The true center of the light distribution will be referred to as the *input* and its estimated center will be referred to as the *output*

Figure 1 shows a flowchart of the simulation. It provides an overview of how a light distribution representing the pupil or the corneal reflection of a light source is modeled and captured on the image sensor, and how the estimated center of this light distribution is computed from the resulting digitized image. The true center of the light distribution will be referred to as the *input* and its estimated center will be referred to as the *output*. To model the light distribution, a 2D Gaussian is generated and digitized on the image sensor, and then noise from another Gaussian distribution is added to the digitized image. Three different versions (called *feature images*) of this image are then generated (yellow boxes in the figure). First, a binary “Top hat” image is created by thresholding. The Top hat models the pupil or CR in an eye image after a threshold operation. Second, depending on whether or not the image sensor can represent the brightest values of the light distribution (i.e., becomes saturated or not), two different types of intensity images are created: a truncated Gaussian representing the saturated case and a full Gaussian representing the unsaturated case. Finally, four different center estimates are provided from the binary and intensity images by computing their center-of-mass (CoM) (paths indicated by ①-③ in the figure) or by computing the center of an ellipse fitted to the contours of the Top hat (path ④). The two latter center localization methods (③ and ④) are commonly used in the literature and commercial systems (e.g., SR Research, 2017; Mulligan, 1997; Li & Pelz, 2006). In the remainder of this paper, these four estimated centers will be referred to as *Gaussian full (CoM)*, *Gaussian Truncated (CoM)*, *Top hat (CoM)*, and *Top Hat (ellipse)*.

To assess the localization accuracy across a range of different input locations, the center of the light distribution used to generate the feature images will be moved horizontally in sub-pixel steps (≪ 1 pixel), which would correspond to very small eye movements (≪ 1 deg) in a typical eye-tracker setup.

### Methods

**Fig. 2:**
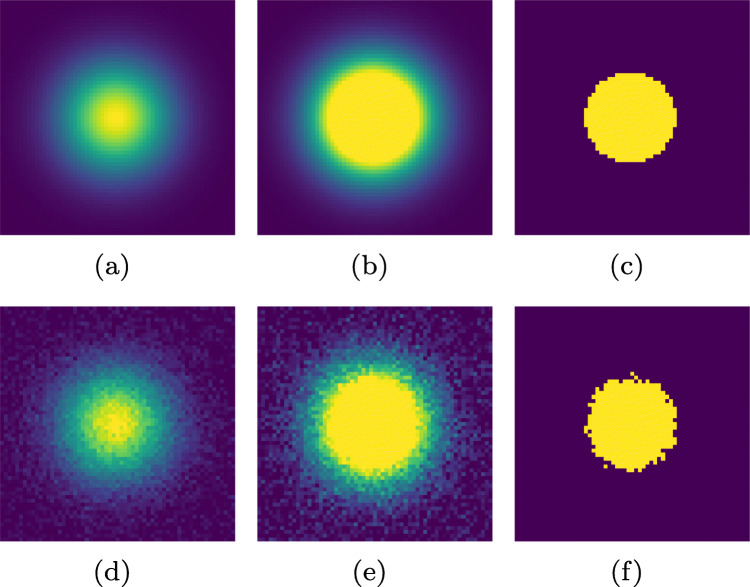
Example of how eye image features (pupil or CR) can be represented in an eye image. A feature originating from a light source where the image sensor is not saturated (*left*), saturated (*middle*), and a binarized version of these (*right*). The features are represented by a full Gaussian (*left*), a truncated Gaussian (*middle*), and a Top hat (*right*). Noise from a Gaussian distribution $\mathcal {N}(0, \sigma ^{2})$, where *σ* = 10 was added to images in the second row (cf. Fig. 1)

More formally, the feature image in Fig. 2a is generated from a Gaussian distribution

1 $$ G(x,y) = \frac{1}{2 \pi {\sigma_{w}^{2}}} e^{-(\frac{(x - x_{c})^{2} + (y - y_{c})^{2}}{2 {\sigma_{w}^{2}}})}  $$

centered at location (*x*_*c*_,*y*_*c*_), which represents the center of the input light distribution, and where *x* ∈{*x*_*c*_ − 3*σ*_*w*_,*x*_*c*_ − 3*σ*_*w*_ + 1,…,*x*_*c*_ + 3*σ*_*w*_ − 1,*x*_*c*_ + 3*σ*_*w*_}, and *y* ∈{*y*_*c*_ − 3*σ*_*w*_,*y*_*c*_ − 3*σ*_*w*_ + 1,…,*y*_*c*_ + 3*σ*_*w*_ − 1,*y*_*c*_ + 3*σ*_*w*_}. In Fig. 2a, this Gaussian is represented by an 8-bit image, where the maximum value corresponds to an intensity value of 255. In Fig. 2b, the 2D Gaussian in (a) has been truncated at half its maximum height (intensity value 128). Finally, the ’Top hat’ in Fig. 2c is represented by the area of the Gaussian in (a) truncated at half its maximum height (intensity value 128) and otherwise zero. To model saturation on the image sensor, the intensity values of the images in Fig. 2b and c were re-scaled to the range [0,255], by dividing each intensity value by the maximum intensity value in the image, and then multiplying by 255. The second row in Fig. 2 illustrates the same features images as in the first row, but after noise was added at the image sensor (cf. Fig. 1).

The center location of each feature is approximated by its center-of-mass (CoM), computed from pixel intensity values in the feature images. The horizontal CoM is formally computed as (Shortis et al., 1994),

2 $$ CoM_{x} = {\sum}_{j=1}^{m} {\sum}_{i=1}^{n} i \cdot I(i,j) / {\sum}_{i=1}^{m} {\sum}_{i=1}^{n} I(i,j) $$

where *I*(*i*,*j*) denotes the pixel intensity value at row *i* and column *j* in an image *I*, and (*m*,*n*) represent the dimensions of the image. Similar computations can be performed for the vertical CoM. The exception is what will be referred to as the ‘Top hat (ellipse)’, which represents the center of an ellipse fitted to contour points extracted from the Top hat. We adopt the terminology of Shortis et al., (1994) and refer to the Gaussian distributions as *intensity features* and the Top hat and Top hat (ellipse) as *binary features*. To investigate how accurately the center of a feature (i.e., pupil or CR) can be estimated across a range of input locations, *x*_*c*_ was moved in steps of *δ*_*x*_ = 0.01 pixels in the horizontal direction over a one pixel range (100 steps), and compared to the output of the four different center estimation methods.

We also simulated how image noise and feature size affected localization accuracy. Image noise was drawn from a Gaussian distribution $X \sim \mathcal {N}(0, \sigma ^{2})$ where *σ* = {0,2,4,…,20}, and was added to pixel intensity values of the feature images, which are restricted to a pixel intensity range of [0,255]. In simulations with noise, each sequence of 100 steps was repeated 100 times to be able to quantify the average impact of the noise. To simulate features of different sizes, we varied the standard deviation of the function *G*(*x*,*y*) in Eq. 1 between *σ*_*w*_ = {2,4,6,…,20} pixels, where larger values correspond to wider distributions and hence larger diameters of the Top hat. All simulations were performed with Python (v. 3.6.9) and OpenCV (v. 3.3.1).

### Results

**Fig. 3:**
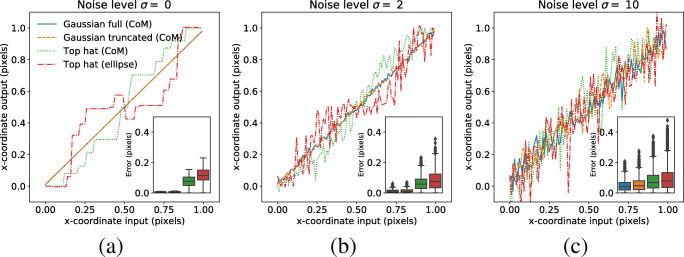
Errors in center localization of an eye image feature (pupil or CR) for different levels of image noise. Values on the *x*-axis represent moving the true center of an input light distribution in steps of 0.01 pixels in the horizontal (*x*) direction (cf. Fig. 1). The output on the *y*-axis represents the estimated center of the light distribution at each step, estimated using one of two methods. The center-of-mass (CoM) is estimated as the weighted average of pixel intensities in a feature image. The ‘Top hat (*ellipse*)’ represents the center of an ellipse estimated from contour points taken from the Top hat. In **a**, no noise was added to the feature images prior to feature center estimation. In **b** and **c**, noise from Gaussian distribution $\mathcal {N}(0, \sigma ^{2})$ was added ((b): *σ* = 2, (c): *σ* = 10). A perfectly accurate center estimation would lead to a straight line with slope 1; this is almost achieved by the Gaussian full (CoM) and Gaussian truncated in (a). All of the results presented here were produced with a fixed feature size corresponding to a Top hat diameter of 14.13 pixels (equivalent to the FWHM of a Gaussian function with *σ*_*ω*_ = 6 in Eq. 2.1). The figure *inset* shows boxplots of the errors between the input and output for each estimated center and step. In case noise was added, the boxplots include all errors across 100 separate simulations

Figure 3a shows the error in feature center localization (output) as a function of the horizontal position of the input, for the different methods to localize the center. Boxplots summarizing the distribution of the absolute error for the different center estimates are shown in the figure inset.

As can be seen from Fig. 3a, the center of the intensity features can be estimated with a high accuracy (low error). Centers estimated from the binary feature are much less accurate, and there are a number of observations that can be made from these estimates: 1) an even step size in the input corresponds to highly uneven steps in the output; sometimes the input steps are not reflected at all in the output, and sometimes they are substantially over-, or underestimated; 2) while the Top hat (CoM) signal always has zero or positive steps, the Top hat (ellipse) signal sometimes has negative steps in response to a positive translation of the input; 3) there is a clear systematicity in how the error unfolds over the one pixel range, where signals are mirrored around the center of the range (input pixel position 0.5). These results were produced with an input light distribution with a size corresponding to a Top hat diameter of 14.13 pixels.

Figure 3b shows that when adding noise with a level of *σ* = 2, the errors in center localization for the intensity features are still small but increase with about a factor two compared to Fig. 3a (cf. figure insets). In contrast, the absolute median error ($\tilde {\epsilon }$) in the Top hat (CoM, $\sigma =0 : \tilde {\epsilon }=0.076$ pixels; $\sigma =2 : \tilde {\epsilon }=0.060$ pixels) and the ellipse fit ($\sigma =0 : \tilde {\epsilon }=0.114$ pixels; $\sigma =2 : \tilde {\epsilon }= 0.075$ pixels) both decrease after noise was added. At noise level *σ* = 10, the differences in localization error across the different center estimates become smaller. While the error for the intensity features continues to increase, the error in the Top hat (CoM) increases compared to noise level *σ* = 2 ($\sigma =10 : \tilde {\epsilon }=0.067$). The error of the ellipse fit remains on a similar level ($\sigma =10 : \tilde {\epsilon }=0.075$). Note that since noise was added, the errors can change somewhat between different simulations. Boxplots in the figure insets represent data from 100 simulations. The curves, however, represent data from one of these simulations for the purpose of illustration.

From Fig. 3, it looks like the total error decreases as more noise (random error) is added. To estimate the systematic component of the error, i.e., the error that remains after removing the influence of random errors, in Fig. 3b and c, we used the average output values across the 100 simulations. The systematic error is then quantified using the same error measure as before, but now using the average value instead of values from an individual simulation. Since no random component is present when *σ* = 0 (Fig. 3a), the systematic error can here be computed directly. The systematic error decreases with increasing noise level for both the Top hat (CoM, $\sigma =0 : \tilde {\epsilon }=0.076$ pixels; $\sigma =2 : \tilde {\epsilon }= 0.049$ pixels; $\sigma =10 : \tilde {\epsilon }= 0.011$ pixels) and the ellipse fit ($\sigma =0 : \tilde {\epsilon }=0.114$ pixels; $\sigma =2 : \tilde {\epsilon }= 0.049$ pixels; $\sigma =10 : \tilde {\epsilon }= 0.006$ pixels).

**Fig. 4:**
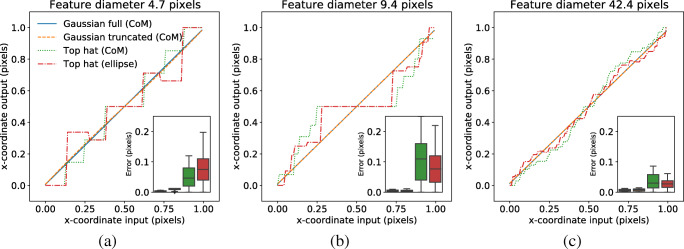
Errors in center localization of an eye image feature (pupil or CR) for different features sizes. Values on the *x*-axis represent moving the center of the input light distribution in steps of 0.01 pixels in the horizontal (*x*) direction. The output on the *y*-axis represents the estimated center of the eye image features at each step, estimated using one of two methods: the center-of-mass (CoM) or fitting an ellipse to contour points taken from the Top hat (Top hat (*ellipse*)). The feature diameter corresponds to the diameter of the Top hat. The figure *inset* shows boxplots of the errors between the input and output for each estimated center and step

Figure 4 illustrates how a larger Top hat diameter leads to increasingly less quantized center estimates for the binary features, whereas the errors from the intensity features are very small irrespective of the feature size. It can also be observed that, unlike estimates for the Top hat (CoM), the ellipse center estimate can provide negative output steps for a positive input step. Note that the error of the Top hat (CoM) in Fig. 4b is larger compared to Fig. 4a, despite the larger feature size in Fig. 4b, meaning that error is not strictly decreasing as a function of feature size. As in Fig. 3, it can clearly be seen that the error repeats when the input moves across a 1 pixel range, such that the signal is mirrored at *x* = 0.5 pixels.

**Fig. 5:**
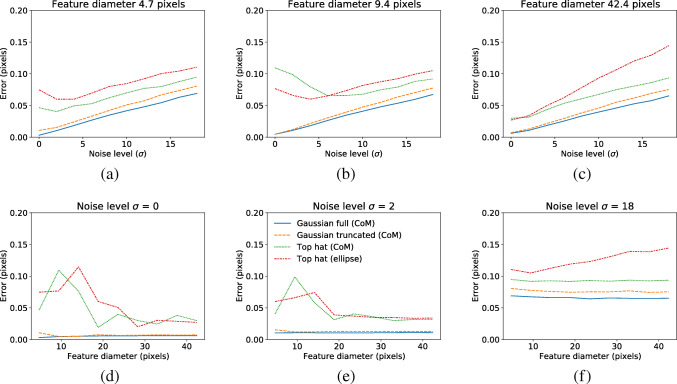
Error in feature center localization using different feature sizes and amount of noise added to the feature images in Fig. 2. The feature size is here represented by the diameter of the Top hat

Figure 5 provides an overview of how the localization error changes as a function feature size and image noise.

An overall observation is that the intensity-based features provide lower errors at low noise levels and for small feature sizes. At higher noise levels and for larger feature sizes there is a smaller advantage of using intensity-based features. The truncated Gaussian generally leads to larger errors compared to using the full Gaussian, but the difference is small.

### Discussion

We conclude that how accurately a subpixel movement of a reference input, e.g., light from a corneal reflection, is represented in a digitized image depends on how the light distribution on the image sensor is represented, where the light distribution is located on the sensor, how many pixels on the sensor the light covers (feature size), how much noise is present in the image, and what method is used to estimate the center of the feature. An important finding is that very small localization errors can be achieved by using a large feature (i.e., pupil or CR) size in combination with low levels of image noise.

We also conclude that, for center estimation methods that rely on binary input (Top hat (CoM), Top hat (ellipse)), there is a systematic error in center estimation as the reference input travels across a one pixel range in the camera image; the error is mirrored around the input position *x* = 0.5. Through noise simulations, it has also become clear that the true center of the light distribution can, for low noise levels, be more accurately estimated using intensity-based features (Gaussian full (CoM), Gaussian truncated (CoM)) compared to binary features (Top hat (CoM), Top hat (ellipse)). The intensity-based features also do not show apparent problems with systematic deviations similar to those reported for the binary features. At high levels of noise the differences in localization error between the binary and intensity-based features become smaller, and the systematic error of the binary features decreases (Fig. 3c). An intriguing result from our simulations for binary features is that—while noise inherently decreases the precision of the output signal and therefore makes it more difficult to detect small steps in the input—large errors can occur also at low noise levels due to systematic deviations. The exact mechanisms by which noise sometimes decreases localization errors and by which larger feature sizes do not always provide smaller errors remain to be investigated.

The results from the simulations provide a potential explanation for the findings reported by Holmqvist and Blignaut (2020) who write that “A major unsolved issue is exactly what it is that causes the errors in the CR signal in modern VOGs”. What they refer to as ‘shrinkage’ and ‘magnification’ in the CR and gaze signals can clearly be seen in our simulations (e.g., Fig. 4), where the same step in the input provides shorter or longer steps in the output depending on where in the one pixel range one is.

## Putting the simulations in an eye tracker context

**Fig. 6:**
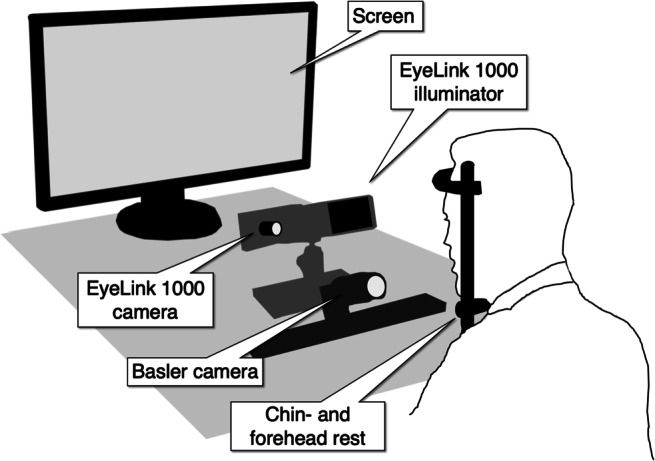
Eye-tracker setups. The EyeLink 1000 Plus setup was used in desktop mode. A Basler camera was placed in front of the EyeLink camera (FLEX setup), such that data could be recorded simultaneously with both setups. The EyeLink illuminator was used for both setups. Only data from the left eye were recorded

While the simulations show how the distribution and size of image features as well as image noise relate to how accurately the features (i.e., pupil and CR) can be localized, it is not clear in what range of the parameter values eye trackers typically operate, and how pixel values relate to degrees of eye rotation. Are these simulations representative for situations one might encounter with an eye tracker? In this section we ask where in the range of the simulations a real eye tracker resides. To address this question, we consider pupil and CR signals extracted from two different setups (Fig. 6): the EyeLink 1000 Plus in desktop mode and FLEX (Hooge et al., 2021), a self-built eye-tracker using a high-resolution (672 × 340 pixels), high-framerate (1000 Hz) camera in combination with a custom made image processing and gaze estimation pipeline.

To investigate what we can expect from these setups based on the simulations in Section “Simulations”, we re-run the simulations using parameters estimated from the two eye-tracker setups. To estimate these parameters, we need to 1) estimate the size (in pixels) of the pupil and CR on the image sensor, 2) estimate the image noise and 3) estimate the relationship between an eye rotation in degrees and the displacement of the pupil/CR on the image sensor. The last step is required to be able to convert the one pixel range from the simulations in Section “Simulations” to an eye rotation (in degrees).

### Participants

The first and second authors of this article participated. They did not wear glasses or contact lenses. Only recorded data from the left eye were considered.

To verify that image noise was not overestimated due to physiological motion of the eye relative to the camera, we also used an artificial eye (provided by SR Research). The study was approved by the Ethical Review Board in Sweden (Dnr: 2019-01081).

### Hardware

The visual stimuli were presented on an ASUS VG248QE screen (531 x 299 mm; 1920 x 1080 pixels; 60-Hz refresh rate), which was placed 80 cm in front of the participant’s eyes. The EyeLink camera and illuminator were positioned 50 cm in front of the participant. A chin-, and forehead rest was used to immobilize participants’ heads. The EyeLink 1000 Plus with the 35-mm lens was used in desktop mode (see Fig. 6). Binocular, uncalibrated data from the EyeLink were acquired at 1000 Hz in pupil centroid mode, and both heuristic filters were switched off. Version 5.12 of the EyeLink host software was used in combination the DevKit 1.11.571, allowing us to record uncalibrated positions and sizes of the pupil and the corneal reflection in the coordinate system of the EyeLink camera.

**Fig. 7:**
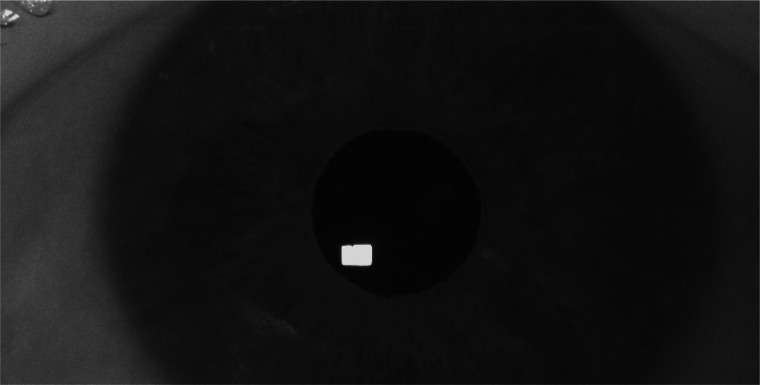
Example eye image from the FLEX setup (resolution 672 x 340 pixels @1000 Hz). The eye is illuminated with the illuminator from the EyeLink setup

In the FLEX, a Basler camera (Basler ace acA2500-60um) was fitted with a 50 mm lens (AZURE-5022ML12M) and a Near-IR Long pass Filter (MIDOPT LP715-37.5). It was placed directly in front of the EyeLink camera as illustrated in the figure, and filmed the left eye. Eye videos were recorded at 1000 Hz at a resolution of 672 x 340 pixels (exposure time: 882 *μ* s, Gain: 12 dB), and encoded as mp4-files at 10-bit luminance resolution using FFmpeg version 4.4 and the libx264 h.264 encoder (preset: veryfast, crf: 0 (lossless), pixel format: yuv444p10). Note that both setups (FLEX and EyeLink) used the EyeLink 890 nm illuminator (at 100% power) to illuminate the eye to provide sufficient contrast between the pupil and iris border as well as generate a reflection on the cornea that can be tracked in the eye image. Recording was done concurrently with both setups. An example eye image recorded with the FLEX setup is given in Fig. 7.

### Image analysis

Video frames captured from the Basler camera were analyzed frame-by-frame at 8 bit luminance resolution to find the pupil and CR centers (Hooge et al., 2021). This was done in three steps. First, thresholds were identified separately for the pupil and the CR through manual inspection of eye images from the beginning of a recording from each participant. Second, the thresholds were used to binarize the eye images and, based on size and shape criteria, detect blobs in the binary images likely to reflect the pupil and the CR regions. Consequently, only the Top hat is considered in the analysis of real eye-tracker data. Third, the center of each blob was localized by computing its center-of-mass. The pupil diameter (*d*) was computed as $d = 2 \sqrt {A / \pi }$, where A is the area of the detected pupil in pixels. Because our model uses a circular CR, we approximated the CR in the camera image with a circular CR that had the equivalent area (number of pixels) as the rectangular CR in the camera image. We determined the diameter of this circular CR using the same formula as above.

### Procedure

The focus of the EyeLink and FLEX cameras were adjusted such that the size of the CR in the left eye was minimized. The participants then performed different tasks, two of which are used in this paper: First, they fixated a blue dot (diameter 0.6 deg) with a red dot (diameter 0.1 deg) in the middle on a gray background located in the center of the screen for 10 seconds. Then, they fixated a dot that was presented at positions (*x*,340),*x* ∈{360,660,960,1260,1560} pixels (with upper left corner as origin), one position at the time. The dot stayed at each position for 1 second. The distance between the dot positions, and hence the target amplitude for each saccade, was equivalent to 5.93 deg.

Finally, we positioned an artificial eye where the participants had their left eye during the recordings, and recorded another 1000 frames from the artificial eye with the FLEX setup. The artificial eye was attached to a piece of cardboard and mounted on the EyeLink chin-, and forehead rest. Before the recording started the artificial eye was manually adjusted to point in the general direction of the screen.

### Data analysis

The recorded data include timestamped pupil and CR positions and sizes, for both setups, as well as eye images for the FLEX setup.

In the simulations in Section “Simulations”, image noise was added to each frame independently. In videos recorded with the FLEX setup here, we cannot directly estimate the image noise in a frame. Instead, subsequent frames were subtracted, and the standard deviation *s* of the resulting pixel intensity differences was computed. Since the variance *σ*^2^ of the difference between two Gaussian distributions with variances ${\sigma _{1}^{2}}$ and ${\sigma _{2}^{2}}$ is

3 $$ \sigma^{2} = {\sigma_{1}^{2}} + {\sigma_{2}^{2}},  $$

the sample variance *s*^2^ needs to be divided by 2 before it can be compared to the variance *σ*^2^ used in the simulations. Consequently, the standard deviation *s* needs to be divided by $\sqrt {2}$. To minimize variation in pixel intensities due to eye movements when estimating image noise, we used 1000 frames (1 s) from a video where the participant had the task to fixate the center of the screen.

To be able to estimate the relationship between an eye rotation in degrees and the displacement of the pupil/CR in the eye image in pixels, participants were asked to perform saccades with known target amplitudes (5.93 deg). Note that the exact target amplitude is not critical for establishing this relationship, even though too small amplitudes may decrease its reproducibility due to a lower signal-to-noise ratio. In Section “Simulations”, the input was moved only along the horizontal dimension of the image sensor. Since this is unlikely the case in our eye-tracker setups, the 2D displacement of the center of an eye image feature (in pixels) as a result of the saccade was estimated manually from horizontal and vertical pupil-, and CR signals by clicking the mouse at the position of the saccade onset and offset. This manual analysis was conducted by the first author.

### Results

The average pupil and CR diameters in the camera image for the recorded data are shown in Table 1. It can be seen that the pupil is larger by more than a factor 3 in the FLEX setup compared to the EyeLink setup (about a factor 2.5 for the CR).

**Table 1:** Pixel displacements of the CR and pupil centers in the eye image corresponding to an eye rotation in response to a 5.93 deg displacement of the stimulus on the computer screen

PID	Setup	Pupil diam. (px)	CR diam. (px)	Disp. Pupil (px)	Disp. CR (px)	*s*
P_1_	EyeLink	46.6 (1.6)	10.2 (0.1)	15.68 (0.44)	8.84 (0.25)	–
	FLEX	147.4 (3.3)	23.5 (0.2)	50.00 (1.31)	29.26 (1.14)	4.12
P_2_	EyeLink	53.8 (1.7)	9.4 (0.1)	18.13 (1.08)	10.67 (0.83)	–
	FLEX	177.4 (5.9)	24.5 (0.3)	58.61 (3.23)	34.73 (2.07)	3.94

‘*s*’ corresponds to the average standard deviation of differences in pixel intensities between consecutive frames in recorded eye videos, and represents the level of images noise. Values for diameter represent median (median absolute deviation in parenthesis) and for displacement mean (standard deviation in parenthesis). Values from two participants (PID) and two eye-tracker setups (FLEX, EyeLink) are provided

**Fig. 8:**
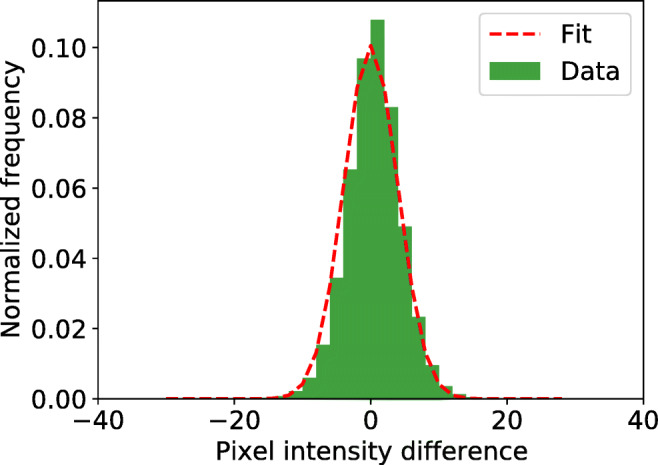
Distribution and Gaussian fit of differences in pixel intensity values between consecutive frames. The plot was generated from 1000 frames recorded with the FLEX setup while participant *P*_2_ was fixating a static target

Figure 8 shows a histogram of pixel intensity differences between frames recorded with the FLEX setup along with a Gaussian fit of the differences.

The estimated standard deviation *s* of the pixel intensity differences is provided in Table 1 (last column) and is around 4 units, thus corresponding to slightly lower standard deviations of $4/\sqrt {2}$ units in the simulations. Temporal image noise estimated from the artificial eye (*s* = 4.24) was similar to that estimated from the two human participants (P_1_: *s* = 4.12, P_2_: *s* = 3.94).

Table 1 also shows the median displacement in the pupil-, and CR-signals as a result of the four saccades conducted by each participant. Using the fact that each saccade had a target amplitude of 5.93 deg, we can from the table compute how many pixels the pupil and CR centers translate in the eye image for a 1 min arc eye rotation. For the EyeLink setup, a 1 min arc eye rotation for P_1_ (first participant) corresponded to a displacement of 0.05 pixels (P_2_: 0.05 pixels) in the pupil signal and 0.03 pixels (P_2_: 0.03 pixels) in the CR signal. For P_1_ in the FLEX setup, the corresponding numbers are 0.14 pixels (P_2_: 0.16 pixels) for the pupil and 0.08 pixels (P_2_: 0.10 pixels) for the CR. Consequently, the table allows us to predict the localization accuracy for the two eye-tracking setups in the context of the simulations, and instead of pixels in the eye image express the results in degrees of eye rotation.

To be able to relate pixels in the eye image to mm in the world, we put a tape measure at the eye position and recorded a frame with the FLEX setup. From this frame, it was computed that 1 mm corresponded to 23.68 pixels. Consequently, to be able to measure eye rotations of 1 min arc from the CR in our FLEX setup, we need to be able to resolve displacements on the image sensor as small as 3.4 *μ* m (0.08/23.68) for P_1_ (P_2_: 4.2 *μ* m, [0.10/23.68]).

**Fig. 9:**
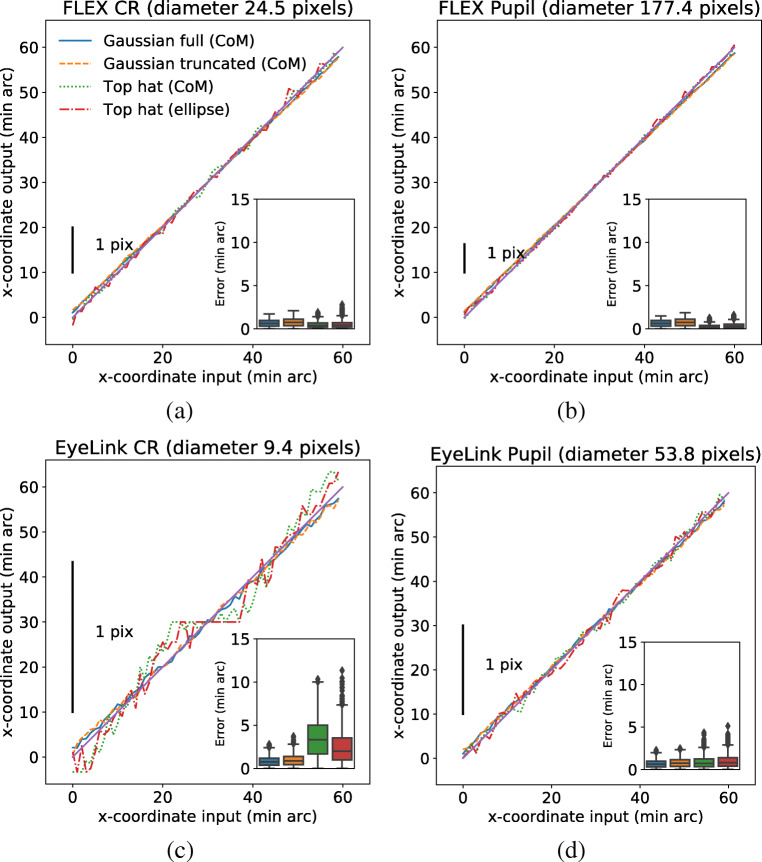
Errors in center localization for the pupil and CR in two eye-tracker setups (EyeLink and FLEX) for participant P_2_. Values on the *x*-axis represent moving the center of the input light distribution in steps of 1 min arc in the horizontal (*x*) direction. The output on the *y*-axis represents the estimated center of the eye image features at each step, estimated using one of two methods: the center-of-mass (CoM) or fitting an ellipse to contour points taken from the Top hat (Top hat (ellipse)). The pupil and CR diameters correspond to the diameter of the Top hat. The figure *inset* shows boxplots of the errors between the input and output for each estimated center and step. The pupil and CR sizes were taken from the EyeLink data files and the output of the image-processing pipeline in the FLEX setup. The amount of image noise was estimated from FLEX eye images (cf. Table 1)

Figure 9 illustrates the results of a simulation where pupil and CR sizes of the FLEX and the EyeLink setups were used. Since no eye images were available from the EyeLink setup, and therefore no image noise estimates can be performed, all simulations were conducted with the noise level $\sigma = s / \sqrt {2}$ estimated from FLEX images recorded from participant *P*_2_ (*cf.* Table 1). The plots illustrate the error in feature localization over the range of 1 degree (60 min arc), where each step corresponds to 1 min arc. To relate the results in Fig. 9 to the simulations in Section “Simulations”, the interval spanning a one pixel range is indicated by a black vertical bar in each plot.

From these plots we conclude that small displacements of the true center of the light distribution (input) are accurately represented in the estimated center of the light distribution (output) of the FLEX setup, regardless of image feature (pupil or CR) or method used for feature localization (binary or intensity based). The error between an input and estimated output is on average less than 1 min arc, and very few errors are larger than 2 min arc. The same applies for the pupil feature in the EyeLink setup, since the size of the pupil is larger than the CR size in the FLEX setup. However, using binary methods for center localization of CR features in the EyeLink setup can give rise to errors in the order of 5 min arc. The largest error is reached when localizing the CR center with the binary Top hat (Fig. 9c, green color). Note that in this case, a large part of the total error seems to be explained by a systematic deviation from the ideal relationship between the input and output (a line with unit slope). Indeed, after reducing the influence of the random contribution to the total error by computing the average error over 100 simulations, the total error is still $\tilde {\epsilon }= 3.62$ min arc.

### Discussion

We have investigated what feature localization accuracy to expect from two real eye-tracker setups (the EyeLink 1000 Plus and the FLEX) based on the simulations from Section “Simulations”. This taught us (Fig. 9) that the pupil signals are likely not the bottleneck in any of the two setups in terms of localization accuracy, since they span enough pixels to resolve even very small eye movements. However, when the CR becomes as small as that found in the EyeLink setup (a diameter of about 9 pixels in the eye image), localization errors in the CR can be up to 10 min arc, at least if binary methods are used for CR localization.

It should be noted that we do not know the level of noise in eye images acquired with the EyeLink system, since these are not available to the end user. Instead, we have used noise estimates from FLEX eye images in the EyeLink simulation. As was found in Section “Simulations”, a lower amount of image noise generally leads to smaller localization errors cf. Fig. 5). However, for certain eye feature sizes, decreasing the image noise may instead increase the total error (cf. Fig. 5b). If the actual noise in EyeLink eye images was higher than in our simulations, it would likely lead to a noisier gaze signal where small saccades would be harder to detect.

According to Holmqvist and Blignaut (2020), the commercial systems they tested had pupil diameters in the range 10–100 pixels and CR diameters spanning 2–15 pixels, i.e., much smaller compared to the FLEX setup, but on par with the EyeLink setup tested here. Under the assumption that some of them use binary methods to localize the pupil and CR centers (paths ③ and ④ in Fig. 1), poor localization accuracy is unsurprising according to our simulations, leading to the problems (Holmqvist & Blignaut, 2020) mention in their paper.

To conclude, the simulations show that the EyeLink 1000 Plus, but not the FLEX, may have a feature center localization problem, which, in the binary case, originates from the small size of the CR on the EyeLink camera sensor. Localization errors in our simulation of the EyeLink can be more than 5 min arc (Fig. 9), which is similar to the errors shown in Fig. 16 of Holmqvist and Blignaut (2020).

## General discussion

We have simulated how accurately an image feature (pupil and CR) can be localized in an eye image with regard to the size and distribution of the feature, the degree of image noise, and the method used to localize the center of the feature. These simulations reveal under what circumstances the amplitude of small eye movements can be reliably estimated. The two most important contributions of this paper are: 1) that the amplitude of arbitrarily small eye movements can be estimated with using the P-CR eye-tracking principle and 2) a possible explanation was provided to why the amplitude of small eye movements may be over-, or under- estimated in commercial P-CR eye trackers (cf. Holmqvist & Blignaut, 2020).

Our simulations show that two aspects are critical to be able to estimate the amplitude of small eye movements. First, image noise needs to be low; too much image noise leads to a lower precision of the pupil and CR signals (and therefore the gaze signal) and hence makes it difficult to distinguish small eye movements from noise in the signal. Second, the size (in pixels) of an image feature (pupil or CR) needs to be large, such that small rotations of the eye lead to translations of the image feature in the eye image. Mulligan (1997) refers to this as the ‘optical gain’, and argues that it is limited by the fact that relevant eye image features (e.g., the pupil) need to be completely contained within the eye image. Consequently, at least in theory, the amplitude of arbitrarily small eye movements can be estimated by decreasing the image noise and increasing the size of the CR and pupil. Given that new cameras with higher image resolution, lower image noise, and higher frame rates frequently become available, it is likely that future video-based eye trackers will be able to push the limits of what is currently possible to measure even further.

Our results also show that if the CR is not binarized in the eye image, but the intensity values of the CR are instead used when localizing its center (paths ① and ② in Fig. 1), it is possible to achieve a high localization accuracy even when the CR is represented with a narrow Gaussian function (corresponding to a small CR size in the binary case). This offers a promising alternative way forward for algorithm developers to improve the data quality of future video-based eye trackers.

It is important to note that the simulations were performed on a single eye image feature, but a P-CR eye tracker typically uses the vector between the pupil and the CR, and therefore results in a noisier signal that any of the two signals (features) themselves. If the noise follows a Gaussian distribution, for instance, the variance of the P-CR signal would be $\sigma _{pup}^{2} + \sigma _{cr}^{2}$, where $\sigma _{pup}^{2}$ and $\sigma _{cr}^{2}$ denote the variance of the pupil-, and CR-signals, respectively. In many cases, however, the pupil spans enough pixels to avoid many of the problems we saw in our simulations, and the feature localization problems can be ascribed to the CR alone, corroborating the findings in Holmqvist and Blignaut (2020). Therefore, our single-feature simulations are representative also for P-CR signals, with the addition that P-CR signals will exhibit a higher degree of noise compared to pupil-, or CR-only signals.

Our simulations also provided an explanation to why many commercial eye trackers have difficulties to accurately estimate the amplitude of small eye movements from the gaze signal (cf. Holmqvist & Blignaut, 2020). When the pupil or CR span few pixels in the eye image, the results from the simulations can be characterized as the ‘one pixel problem’, meaning that the displacement of an eye image feature in response to a very small eye rotation is influenced by where this feature is on the camera sensor. As a consequence, the same eye rotation may lead to differently estimated displacements of the CR, and even displacement in the opposite direction of the eye rotation. Given the CR sizes reported in Holmqvist and Blignaut (2020), the ‘one pixel problem’ seems to be a plausible explanation to why the same physical movement of an artificial eye is sometimes ‘shrunk’ or ‘magnified’.

So are all the problems with P-CR eye trackers solved by using cameras with higher resolution and less images noise? The answer is unfortunately *no*. Other problems that may interfere with the estimation of small saccade amplitudes have been described in the literature. Examples include the pupil size artifact (Wyatt, 2010; Wildenmann & Schaeffel, 2013; Choe et al., 2015; Hooge et al., 2019; Hooge et al., 2021), head motion (Hermens, 2015), pupil foreshortening (Hayes & Petrov, 2016), and issues related to corneal topology (Nitschke et al., 2013). Other practical issues such as real-time image processing and robust center estimation of the pupil and CR in real eye images also need to be considered in practical implementations of eye-trackers that aim to estimate the amplitude of very small saccades.

In conclusion, the P-CR eye-tracking principle can be used to correctly estimate the amplitude of small eye movements given that certain design criteria are fulfilled. Our simulations provide insight into these criteria, and help explain problems current commercial eye tracker have with estimating the amplitude of small eye movements.

## Footnotes

1 VOG refers to video-oculography, which is another name for video-based eye-tracking.
